# Inoculation in Rubeola

**Published:** 1851-03

**Authors:** John E. McGirr

**Affiliations:** Professor of Chemistry, Physiology, &c., in the University of St. Mary’s, Physician to the Catholic Male and Female Orphan Asylums, Chicago


### ARTICLE VIII.

*Inoculation in Rubeola.* By JOHN E. MCGIRR, A. M., M. D., L. L. D., Professor of Chemistry, Physiology, &c., in the University of St. Mary's, Physician to the Catholic Male and Female Orphan Asylums, Chicago.

Inoculation in Rubeola is no new experiment. As to the advantage of the process, diversity of opinion exists. Drs. Home, in Edinburg, Dewees, and Chapman, at the Dispensary in Philadelphia in 1801, practiced inoculation without any satisfactory results, while the experiments of Prof. Speranza of Mantua, and others, were varied, decisive and successful. Having no opinion of my own to confirm, wishing only to arrive at the truth, if possible, I determined when the very favorable opportunity presented, by the breaking out of Rubeola in these Asylums, to test this point. The Asylums are situated, (the female in north, and the male in south Chicago,)

without the thickly settled portion of the city, having the advantage of healthy locations. The houses are large, well ventillated, and are under the charge of the Sisters of Mercy; thus the best nursing could be secured, and the best opportunity which might ever again occur to me of watching every stage of the progress of the disease. Early in December the first case of measles was brought into the female asylum. I proceeded to inoculate from this case, when the eruption was at its height. Blood was drawn from a vivid exanthematous patch on the diseased child's arm, and inserted into the arms of the three children first mentioned in the list below. On the fourth, sixth and seventh days, after the inoculation, the measles appeared, pursuing a regular and mild course. The result of these cases determined me to carry the experiment farther, and that the trial might be a fair one, I selected for comparison those whose physical conformation and constitutional idiosyncracy, seemed most nearly alike, giving the disadvantage of age to the inoculation. The following table contains the names, ages, and results of all the cases whether inoculated or not:

| NOT INOCULATED. | | INOCULATED. | |
|---|---|---|---|
| Died. | Age. | Recovered. | Age |
| Ellen Brown, | 3 y'rs. | Ellen Kehoe, | 11 y'rs. |
| Katy Russell, | 2 | Ellen Grant, | 4 |
| Philomena Kehoe, | 3 | Mary M'Carty, | 8 |
| Elizabeth Patton, | 2 | Rose Mack, | 5 |
| Ellen Crowly, | 5 | Mary Grant, | 9 |
| Recovered. | | Eliza Hurley, | 4 |
| Mary Carroll | 9 | Ann Cahill, | 8 |
| Ann Brennan, | 6 | Ella Welsh, | 5 |
| Mary Patton, | 7 | Ann Mulhall, | 9 |
| Johanna Cahill, | 5 | Ann Hagan, | 3 |
| Emeline Hurley, | 4 | Mary Mulhall, | 4 |
| Mary Nugent | 5 | Ellen McCarty, | 10 |
| Mary Brain, | 10 | Anna O'Brien, | 13 |
| Elvira Gilmartin, | 5 | Cath. Power | 9 |
| Fanny Mooney, | 12 | | |
| Mary Ann Tell, | 10 | | |

This table gives us 29 names, 24 recoveries and 5 deaths, all occuring among those not inoculated. The cases of all those inoculated, commencing from the fourth to the ninth day after inoculation, proceeded regularly, with the ordinary symptoms of simple measles, to convalescence, which was speedy and complete, with one exception viz, the first case. This child entered the asylum about a year ago, suffering with violent ophthalmia. She had been cured. On the disappearance of the measles, the ophthalmia returned, and though the sight was much endangered, yet there now only remains a little weakness which is disappearing. All these cases occurred consecutively from the first week of December to the second week of January.

Four children who were known to have had measles in the spring of 1850, were inoculated; nothing else was observed than the inflammation which would follow any ordinary lancet puncture.

Of those not inoculated with four exceptions, the antecedent symptoms were very severe. The fever was violent; distressing vomiting occurred in three cases. The catarrhal symptoms were violent; throat sore, hoarseness, rigors, cough almost continuous, dry, the whole chest sore, difficult respiration, delirium at night in some of the cases.

Four had the "congestive modification," the eruption appeared slowly and imperfectly; one of these died. Two others presented the Typhoid variety; one died of diarrhea, the other recovered, but afterwards four dangerous ulcerations appeared on the limbs, and gangrenous stomatitis, in the left lower jaw. All of the teeth of that part of the jaw, fell out, the left side of the tongue and the cheek were involved in the disease. This case ultimately recovered. Bronchitis supervened in six cases. Three had partial aphonia, one complete; this one died.

When these last mentioned cases attempted to swallow any liquid, it was thrown back through the mouth and nose with violent expulsiv effort.

In the male Asylum, there were 23 cases and 6 deaths. None were inoculated, but 3 of the whole number had the disease mildly, and these were the three first attacked. The others had violent antecedent symptoms, and tedious convalescence. Five of those who died had the aphonia and difficult deglutition before spoken of, the other died of Phthisis.

In review of these facts much might be said. I have chosen, however, to give them as they occurred, without comments, leaving to the readers of the Journal, to estimate them at what they are worth; merely adding, *that if there is no advantage in inoculation, the result which the second column furnishes, would be a strange anomaly.*

